# CEP164C regulates flagellum length in stable flagella

**DOI:** 10.1083/jcb.202001160

**Published:** 2020-11-09

**Authors:** Madison Atkins, Jiří Týč, Shahaan Shafiq, Manu Ahmed, Eloïse Bertiaux, Artur Leonel De Castro Neto, Jack Sunter, Philippe Bastin, Samuel Dale Dean, Sue Vaughan

**Affiliations:** 1 Biological and Medical Sciences, Oxford Brookes University, Oxford, UK; 2 Trypanosome Cell Biology Unit and Institut National de la Santé et de la Recherche Médicale U1201, Institut Pasteur, Paris, France; 3 Sorbonne Université école doctorale complexité du vivant, Paris, France; 4 Warwick Medical School, University of Warwick, Coventry, UK

## Abstract

Cilia and flagella are required for cell motility and sensing the external environment and can vary in both length and stability. Stable flagella maintain their length without shortening and lengthening and are proposed to “lock” at the end of growth, but molecular mechanisms for this lock are unknown. We show that CEP164C contributes to the locking mechanism at the base of the flagellum in *Trypanosoma brucei*. CEP164C localizes to mature basal bodies of fully assembled old flagella, but not to growing new flagella, and basal bodies only acquire CEP164C in the third cell cycle after initial assembly. Depletion of CEP164C leads to dysregulation of flagellum growth, with continued growth of the old flagellum, consistent with defects in a flagellum locking mechanism. Inhibiting cytokinesis results in CEP164C acquisition on the new flagellum once it reaches the old flagellum length. These results provide the first insight into the molecular mechanisms regulating flagella growth in cells that must maintain existing flagella while growing new flagella.

## Teaser

Stable eukaryotic flagella can maintain their length, and many protists grow new flagella while maintaining existing flagella. Mechanisms regulating this are not well understood. Atkins et al. show that CEP164C is important for this regulation, and these findings provide key novel insights into this process.

## Introduction

Cilia and flagella are highly conserved microtubule-based organelles that have important roles in cell motility and sensing. They can be highly dynamic and short lived, such as primary cilia or those in *Chlamydomonas reinhardtii* (Marshall and Rosenbaum, 2001), or very stable and long lived, such as those in spermatozoa (San Agustin et al., 2015), photoreceptors (Jiang et al., 2015), or the flagella of many protists. Although there is a wide variation in length among cell types, there is generally a defined length for a given cell type (Broekhuis et al., 2013). Many unicellular flagellated and ciliated organisms have an additional challenge, as they must maintain flagella/cilia at a defined length and simultaneously assemble new flagella/cilia in the same cell. This phenomenon has been largely overlooked, and the underlying mechanisms remain unknown. A grow-and-lock model was proposed for the maintenance of stable flagella in which a molecular lock is applied to prevent flagellum length change after assembly (Bertiaux et al., 2018). The molecular mechanisms of how this lock operates are unknown but are important for cells where an existing flagellum must be maintained during assembly of a new flagellum.

Cilia and flagella are composed of a microtubule-based axoneme, which grows from cytosolic basal bodies and extends out of the plasma membrane. They do not contain ribosomes, and all components are synthesized in the cytosol and transported within the organelle via a specialized transport mechanism called intraflagellar transport (IFT; Marshall and Rosenbaum, 2001). The transition zone and transition fibers are situated at the boundary between the axoneme and basal bodies and have been shown to be important for allowing selective entry of molecules into cilia/flagella (Gonçalves and Pelletier, 2017). Trypanosomes are pathogenic protists that have a single flagellum, which remains assembled throughout the cell cycle, with a new flagellum assembling alongside (Kohl and Bastin, 2005). This organism therefore provides an excellent model to study differential regulation of flagellum growth in a single cell. Here, we show that CEP164C is important for the locking mechanism, and our results illustrate a novel concept in the regulation of flagella growth for cells with stable flagella that need to maintain the length of existing flagella during growth of new flagella.

## Results and discussion

### CEP164C is recruited to transition fibers in the third cell cycle after basal body formation

**Figure 1.**
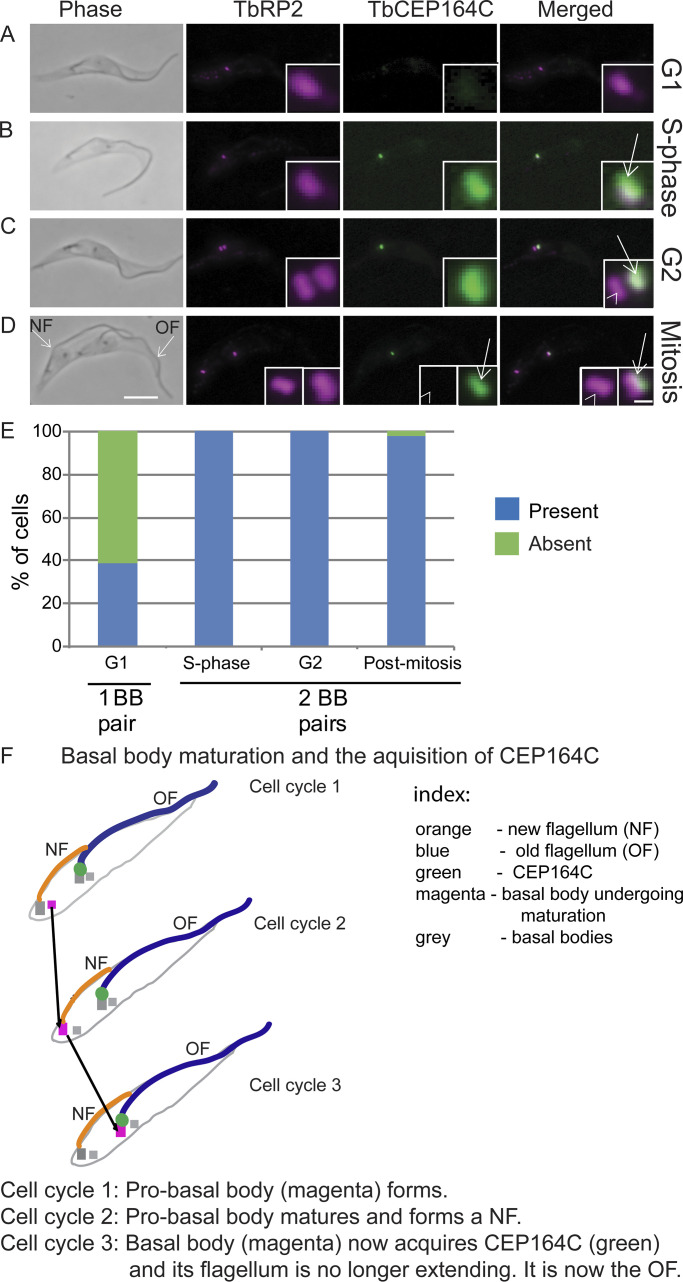
**Cell cycle–dependent localization of CEP164C.** **(A–D)** Endogenously tagged mNG:CEP164C (green) colocalized with transition fiber protein RP2 (magenta) in detergent-extracted cytoskeletons. Scale bars, 5 µm (inset, 400 nm). **(E)** Presence (blue) and absence (green) of CEP164C through the cell cycle; *n* = 200 cells/cell cycle stage). **(F)** Basal body maturation and acquisition of CEP164C acquisition. A basal body is highlighted in magenta to illustrate maturation over three cell cycles before CEP164C acquisition. Arrow, mature basal body of the old flagellum; arrowhead, mature basal body of the new flagellum. BB, basal body; NF, new flagellum; OF, old flagellum.

Recent worked showed that assembled flagella are prevented from further elongation via a lock mechanism (Bertiaux et al., 2018). In mammalian cells, the centrosome protein (CEP) 164 is located at the distal appendages of centrioles and is important for the docking of centrioles to the plasma membrane for assembly of cilia (Graser et al., 2007; Slaats et al., 2014; Čajánek and Nigg, 2014). Given its specific localization, CEP164 could play a role in regulating entry of components into the flagellum and a genome-wide localization study (Dean et al., 2017) showed CEP164C was located only on the old flagellum in biflagellate dividing cells. TbCEP164C was endogenously tagged with mNeonGreen (mNG) at its N-terminus (Fig. 1, A–D) and colocalized with an antibody to the transition fiber protein retinitis pigmentosa-2 (Stephan et al., 2007). In dividing cells, there are two flagella, one old flagellum assembled in a previous cell cycle and one growing new flagellum. CEP164C was only located on the mature basal body of the assembled old flagellum (Fig. 1, C and D, arrow) and not the mature basal body of the new flagellum (Fig. 1, B–D, arrowhead), even though both mature basal bodies were docked to the plasma membrane. Thus, at cytokinesis, the daughter cell with the old flagellum had a CEP164C signal, but the daughter cell with the new flagellum did not (Fig. 1 D). Given this, there should be 50% of G1 cells positive for CEP164C and 50% negative for CEP164C. However, multiple experiments revealed no more than 40% of G1 cells with CEP164C, suggesting that CEP164C is removed from the old flagellum after cytokinesis and then returns at the start of the next cell cycle. (Fig. 1 E; *n* = 800 cells). Removal after cytokinesis could indicate that CEP164C is not needed, as G1 cells are not growing a new flagellum and a lock would only be required when the cell grows another flagellum.

Basal bodies and centrioles have a defined maturation lineage across multiple cell cycles and these results reveal a novel localization pattern that distinguishes the two mature basal bodies in a dividing cell. After initial formation in one cell cycle, a pro-basal body/pro-centriole must proceed through a second cell cycle to mature and assemble a flagellum/cilium. Here, we have shown that it is only in the third cell cycle that CEP164C is acquired, after completion of flagellum assembly in trypanosomes (Fig. 1 F).

### Knockdown of CEP164C leads to dysregulation of flagellum growth

**Figure 2.**
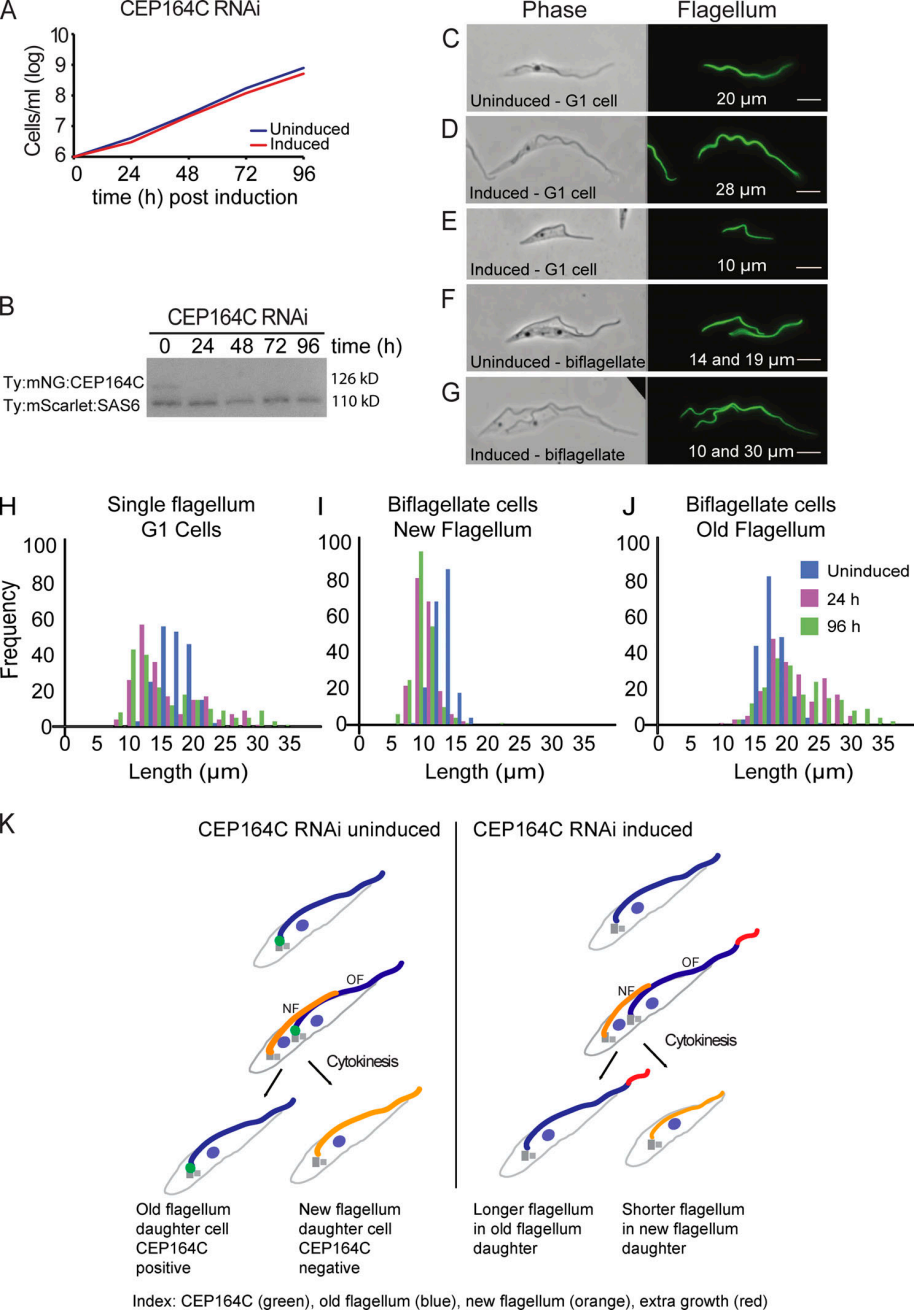
**Knockdown of CEP164C disrupts flagellum length.** **(A)** Growth curve. **(B)** Western blot. Anti-Ty1 antibody (BB2) for knockdown of Ty:mNG:CEP164C (128kD). Ty:mScarlet: TbSAS-6 (110kD) loading control. **(C–G)** Detergent-extracted cells with flagellum marker L8C4. G1 cells with one flagellum (C–E) and biflagellated cells (F and G) are shown. Scale bars, 5 µm. **(H–J)** Measurements of flagellum length in the uninduced condition and 24 and 96 h after RNAi induction. **(H)** Single-flagellum G1 cells (Wilcoxon test 0 h versus 96 h, P = 5.2e-08). **(I)** Postmitotic two-flagella cells (new flagellum; Wilcoxon test 0 h versus 96 h, P = 2.07e-56). **(J)** Old flagellum (Wilcoxon test 0 h versus 96 h, P = 2.024e-10). *n* = 200 cells/time point. **(K)** Absence of CEP164C leads to dysregulation of flagellum growth. NF, new flagellum; OF, old flagellum.

**Figure 3.**
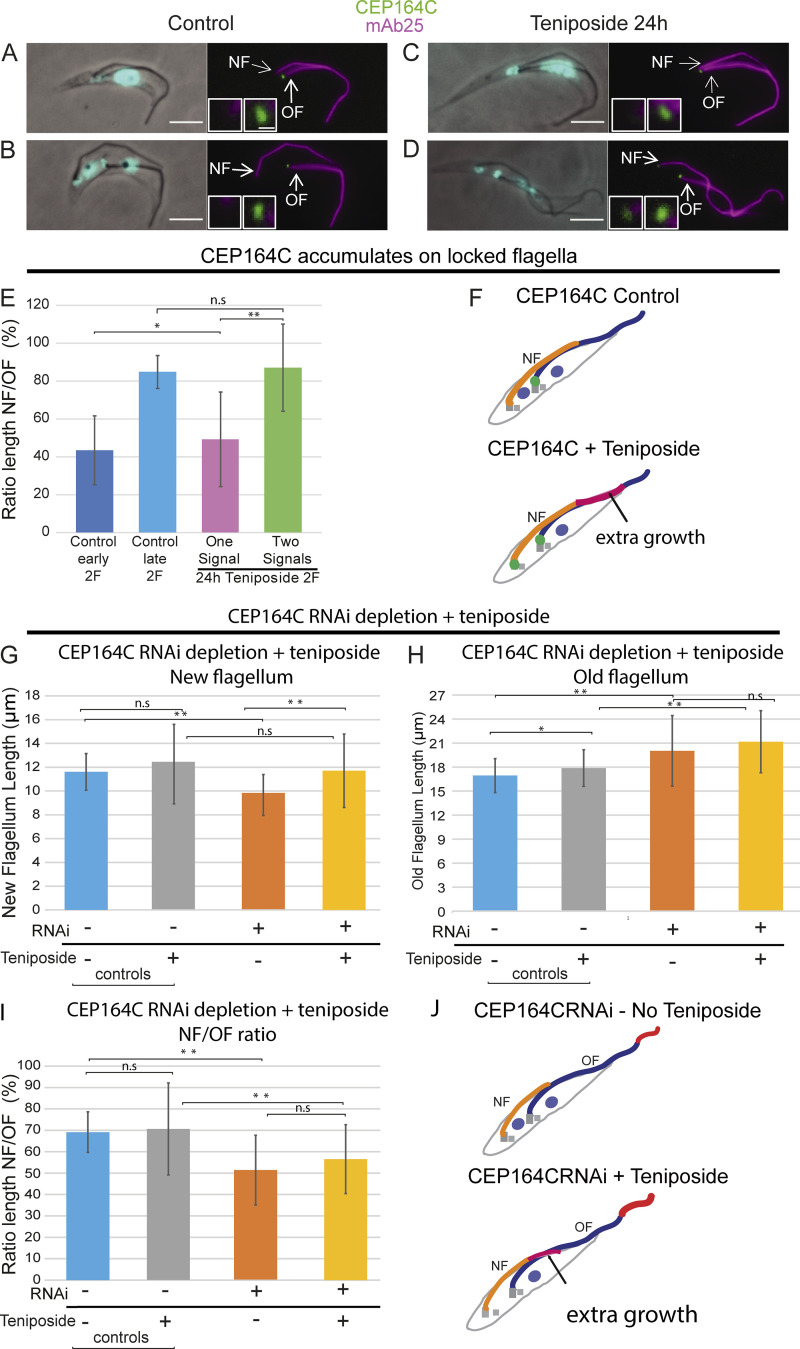
**CEP164C accumulates on locked flagella.** **(A and B)** Untreated cells. mNG:CEP164C is present on the old flagellum (magenta) mature basal body (green) of biflagellated dividing cells early in the cell cycle (A) and after mitosis (B). **(C)** Teniposide-treated cells. mNG:CEP164C is present on the old flagellum (one signal). **(D)** mNG:CEP164C is present on old and new flagella (two signals). **(E)** New flagellum/old flagellum ratios for teniposide experiment. Premitotic control cells, dark blue; postmitotic cells, light blue; teniposide-treated cells with one CEP164C signal, magenta; and teniposide-treated cells with two CEP164C signals, green (*n* = 103 cells). **(F)** Carton illustration of extra new flagellum growth with and without teniposide. New flagellum grows longer in teniposide-treated cells, and CEP164C is acquired on the new flagellum as well as the old. **(G)** CEP164C RNAi + teniposide new flagellum lengths (*n* = 56 cells). **(H)** CEP164C RNAi + teniposide old flagellum lengths (*n* = 56 cells). **(I)** CEP164C RNAi + teniposide new flagellum/old flagellum ratio (*n* = 56 cells). Error bars indicate the variability of the data. **(J)** Cartoon showing a long old flagellum and short new flagellum in CEP164C RNAi without teniposide (top). Following addition of teniposide, there was extra growth on the new flagellum. Wilcoxon test was performed on all datasets. Scale bars, 5 µm. NF, new flagellum; n.s., not significant; OF, old flagellum.

We hypothesized that if CEP164C does play a role in locking further growth of the assembled old flagellum, then depletion would result in dysregulation of flagellum growth on both the old and new flagella. CEP164C knockdown was performed following stable transformation of trypanosomes with a plasmid expressing double-stranded RNA under tetracycline-inducible promotor control (Inoue et al., 2005). Depletion of CEP164C was confirmed by Western blotting, and there was no effect on cell growth or cell motility (Fig. 2, A and B). However, in cells with a single flagellum, flagella were either much longer (Fig. 2 D) or much shorter (Fig. 2 E) than in control cells (Fig. 2 C). Growth of flagella occur during the cell division cycle, with a new flagellum growing alongside an old assembled flagellum, so biflagellated postmitotic cells were investigated to see if there were problems with flagellum growth. In wild-type postmitotic cells, the growing new flagellum is normally slightly shorter than the old flagellum, with assembly completed following cytokinesis (Farr and Gull, 2009). However, there was a significantly greater disparity between new and old flagellum lengths in postmitotic cells in the RNAi-induced population (Fig. 2, F and G); very long flagella were always the old ones (Fig. 2, G and J), whereas the short flagella were always the new ones (Fig. 2, G and I). Measurements of flagellum length in G1 cells with a single flagellum (Fig. 2 H) and dividing postmitotic cells (Fig. 2, H–J) revealed a significantly wider variation in the length of flagella after RNAi induction. In induced postmitotic cells, the old flagellum could reach up to 37 µm in length compared with a maximum of 26 µm in uninduced cells (Fig. 2 J). This disparity in flagellum lengths results in a significantly lower new flagellum/old flagellum ratio at 57% (Fig. 3 I, orange) in RNAi-depleted cells compared with the control at 70% (Fig. 3 I, blue). This confirms that the wide disparity in flagellum lengths seen in G1 cells derived from dysregulation of flagellum growth between the two flagella during the cell cycle. This observation is consistent with CEP164C contributing to a locking mechanism that is lost on depletion of CEP164C, causing continuous flagellum growth of the old flagellum over several cell cycles.

**Figure S1.**
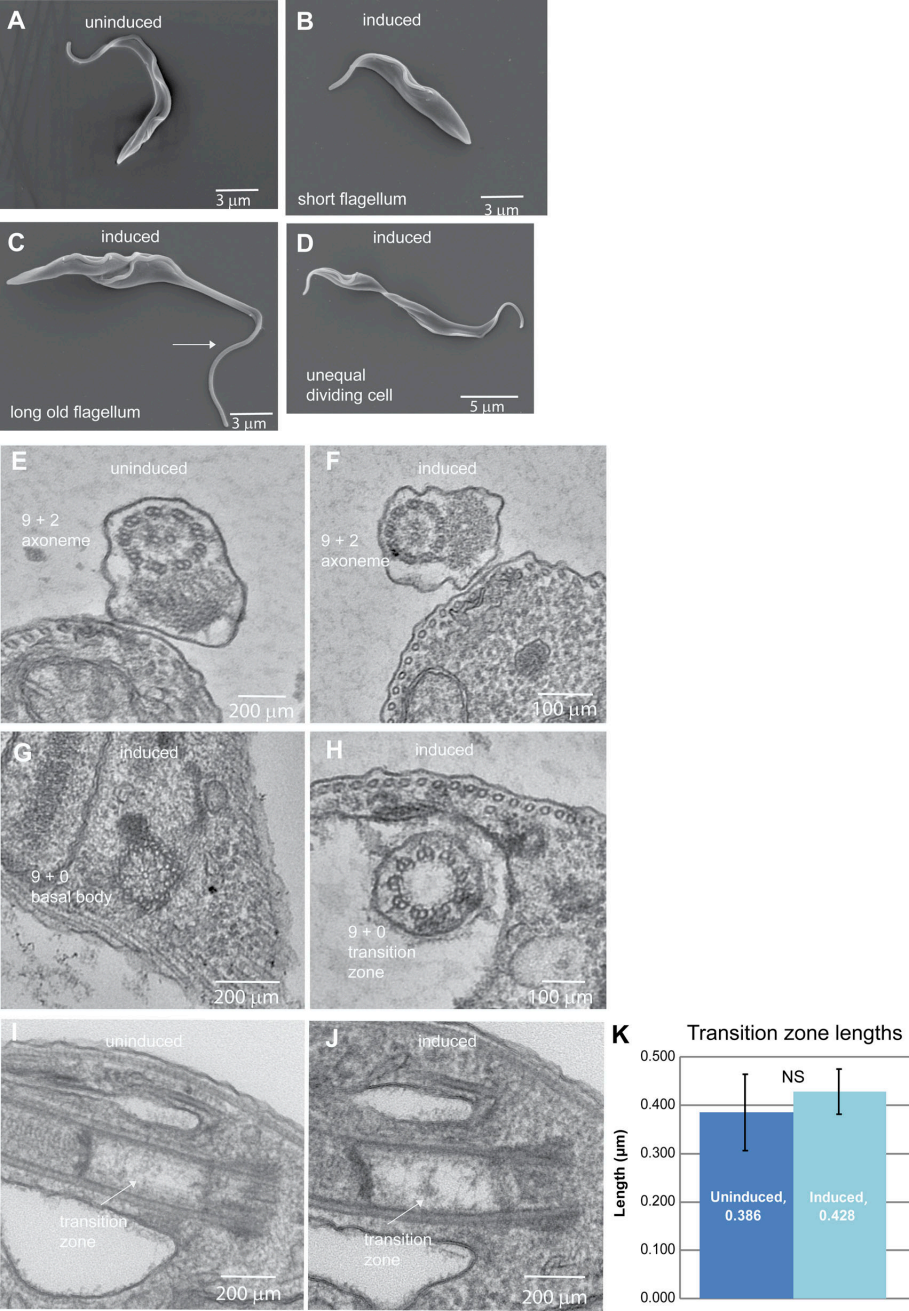
**Ultrastructural analysis of CEP164C RNAi cell line. (A–D)** Scanning EM of uninduced (A) and induced CEP164C RNAi cells (B–D) at 72 h after induction of RNAi illustrating the presence of small cells (B), dividing cells with a long old flagellum (C, arrow), and a dividing cell producing one short and one long daughter (D). **(E–J)** Transmission EM of CEP164C RNAi cells showing cross sections of the 9 + 2 microtubule axoneme in uninduced (E) and induced (F) cells, cross sections of a basal body (G) and a transition zone (H) of induced cells, and longitudinal sections of the transition zone in uninduced (I) and induced (J) cells. **(K)** Measurement of the length of the transition zone in longitudinal transmission EM sections (*n* = 25). Error bars show variability of data.

To determine if IFT differed following CEP164C depletion, IFT52:mNG fusion protein was expressed from its endogenous locus in the inducible CEP164C RNAi cell line. There was no significant difference in the frequency of IFT trains between uninduced (0.57 trains/s) and 72 h after induction (0.55 trains/s; *n* = 10 cells per experiment). Cell shape and ultrastructure of the flagellum were analyzed for potential abnormalities that might cause dysregulation. Scanning EM illustrated normal fusiform cell shape, but with shorter and longer flagella compared with uninduced cells (Fig. S1, A–D). Transmission EM did not reveal any visible abnormalities in the ultrastructure of the flagellum (Fig. S1, E and F) or basal bodies (Fig. S1, G and H). Longitudinal sections of the transition zone were analyzed and measured, with no abnormalities observed and no difference in length seen (Fig. S1, I–K; *n* = 25 sections). The grow-and-lock model predicts that a molecular lock is applied to prevent flagellum length change after assembly, locking the old flagellum into a stable configuration, but this is clearly absent in these induced RNAi cells. Importantly, since there are no abnormally long new flagella in dividing cells, then the abnormally long old flagella must occur via their continued growth in preceding cell cycles when the lock has not been applied (Fig. 2 K).

### CEP164C accumulates on locked flagella

Previous work showed that when the cell division inhibitor teniposide was applied, the new flagellum could grow to the same length as the old flagellum but never exceeded it. This work demonstrated that the new flagellum was locked to prevent further growth even in the absence of cytokinesis, showing that locking of growth is cell cycle independent (Bertiaux et al., 2018). We reasoned that if CEP164C is involved in the locking mechanism, then CEP164C would accumulate at the base of a new flagellum once it was close to the length of the old flagellum in these teniposide-treated cells. To test this, we added teniposide to cells expressing the endogenously tagged mNG:CEP164C. In untreated cells, CEP164C was only at the base of the old flagellum in biflagellated dividing cells, as expected (Fig. 3, A and B). After 24 h of teniposide treatment, CEP164C was still at the base of the old flagellum in biflagellated dividing cells (Fig. 3 C; one signal), but CEP164C was also at the base of the new flagellum in 40% of biflagellated dividing cells (Fig. 3 D; two signals). (Note that this an asynchronous cell population so would only expect a proportion of cells to have longer new flagella and therefore an additional CEP164C signal.) New flagella in cells with one CEP164C signal were significantly shorter than new flagella in cells with two CEP164C signals, resulting in a new flagellum/old flagellum ratio of only 46% in one-signal cells compared with 82% in two-signal cells (Fig. 3 E, dark blue [control] versus one signal [pink]). This would be expected, as the new flagellum in one-signal cells is still growing and no lock has been applied. The two-signal cells have a new flagellum/old flagellum ratio that is almost identical to that of control cells without the addition of teniposide (Fig. 3 E, control late [postmitotic]), suggesting that a lock has been applied in order to prevent further growth. This supports the hypothesis that CEP164C is added to the base of long new flagella in teniposide-treated cells and is involved in the locking mechanism (Fig. 3 F). Finally, CEP164C RNAi-depleted cells were incubated with teniposide to investigate if the short new flagella in CEP164C RNAi-depleted biflagellated dividing cells could grow any longer following addition of teniposide when cytokinesis was inhibited. CEP164C RNAi cells were induced for 24 h, and then teniposide added for 24 h. This showed that the new flagellum grew significantly longer following addition of teniposide (Fig. 3, G and J). The old flagellum did not grow significantly longer (Fig. 3 H), but the new flagellum/old flagellum ratio remained significantly lower in induced CEP164C RNAi cells than in controls, demonstrating that there was continued dysregulation in flagellum growth (Fig. 3, I and J).

### Appearance of ectopic CEP164C on the new flagella basal body

**Figure 4.**
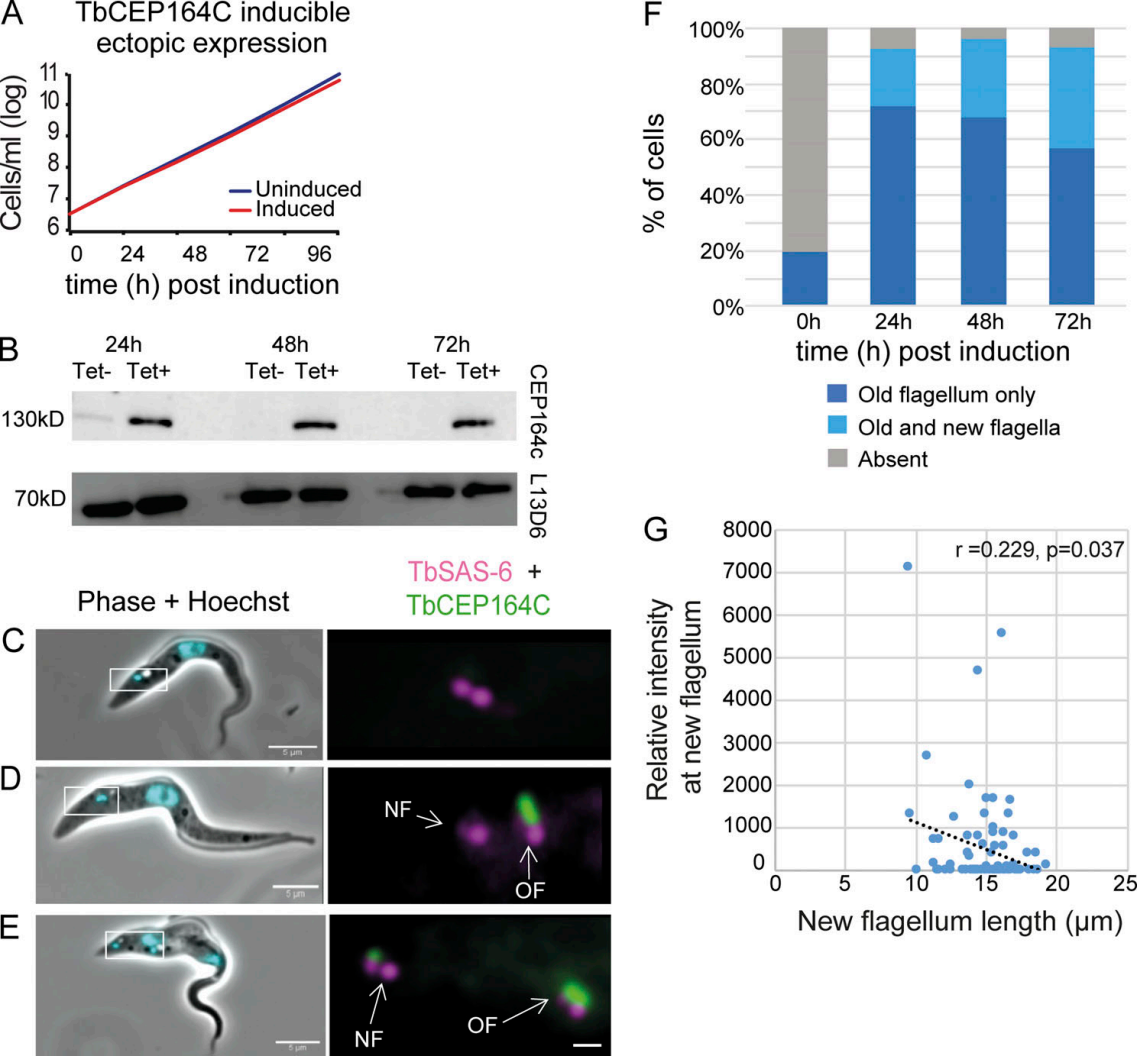
**Inducible ectopic expression of CEP164C.** **(A)** Growth curve. **(B)** Western blot showing inducible expression of ectopic mNG::CEP164C. L13D6 antibody was used as a loading control. **(C–E)** Localization of ectopically expressed CEP164C::mNG (green) after 24 h, endogenously expressed mScarlet:TbSAS6 (magenta) as basal body marker. **(C)** Uninduced cells (mScarlet:TbSAS6 only). **(D)** Induced biflagellate (CEP164C on old flagellum [green]). **(E)** Induced cell biflagellate (CEP164C on the new flagellum and old flagellum). **(F) **Percentage of postmitotic biflagellated cells with ectopic CEP164C (*n* = 200 cells/time point). **(G)** Relative fluorescence intensity of ectopically expressed CEP164C at the new flagellum basal body versus new flagellum length in postmitotic biflagellaged cells (*n* = 84 cells, Pearson’s correlation *r* = 0.229, P = 0.037). Scale bars, 5 µm. NF, new flagellum; OF, old flagellum; Tet, tetracycline.

Given that loss of CEP164C leads to continued growth of the old flagellum, we decided to test if ectopic CEP164C expression caused inappropriate accumulation of CEP164C at the new flagellum basal body leading to dysregulation of flagellum elongation. Inducible ectopic expression of CEP164C::mNG was confirmed by Western blot and had no effect on cell growth (Fig. 4, A and B). Ectopically expressed CEP164C::mNG was absent in most G1 cells with a single flagellum (Fig. 4 C) and localized to the base of the old flagellum in all biflagellated cells, illustrating the same localization pattern as that observed in endogenously expressed CEP164C (Fig. 4 D, OF). However, CEP164C::mNG also located to the base of the new flagellum as well as the old flagellum in 36% of biflagellated cells by 72 h after induction, though often with a lower signal intensity than on the old flagellum (Fig. 4, E and F; *n* = 200 cells). We hypothesized that the new flagellum could be shorter in cells with ectopic CEP164C signal on the new flagellum, as a lock to growth could be applied. To test this, CEP164C signal intensity at the new flagellum base was plotted against new flagellum length. This revealed a trend for shorter flagellum lengths in cells with brighter signal intensity of ectopic CEP164C::mNG (Fig. 4 G; *n* = 84 cells, Pearson correlation, *r* = 0.229, P = 0.037). In summary, these results show that ectopically expressed CEP164C::mNG can abnormally associate with the new flagellum basal body in some dividing cells in addition to its expected localization on the old flagellum basal body. This might occur when all the sites for CEP164C on the old flagellum basal body are occupied, but exactly how proteins are able to distinguish between old and new flagella is still an unanswered question.

### Maintaining an old flagellum while growing a new flagellum

A number of models propose how flagellum length is controlled in eukaryotic organisms, including balance point (Marshall and Rosenbaum, 2001; Marshall et al., 2005), limiting pool (Goehring and Hyman, 2012), cargo loading (Craft et al., 2015), and grow and lock (Bertiaux et al., 2018; Bertiaux and Bastin, 2020). Flagellum assembly has been well characterized in *C. reinhardtii*, where two new flagella are formed simultaneously at each cell cycle and the balance point model predicts that there is an equilibrium between assembly and disassembly with equal access to both flagella. Trypanosomes and other protists must maintain one or more flagella while growing new ones, sometimes over several cell cycles (Bertiaux and Bastin, 2020). In trypanosomes, IFT is not required for maintenance of assembled flagella, as knockdown of IFT components does not shorten an assembled flagellum and increased IFT does not lengthen an already assembled flagellum (Bertiaux et al., 2018). Depletion of CEP164C by RNAi led to further growth of the old flagellum, presumably due to the absence of a lock to growth. Significantly, the new flagellum of the same cell turned out to be shorter. One explanation could be that the new flagellum was unable to reach the normal length as material was also entering the old flagellum, suggesting competition between the two flagella for an intrinsic limit of flagellum components within a cell cycle. Indeed, the soluble pool of tubulin and other flagellar components is small in trypanosomes (Bastin et al., 1998; Schneider et al., 1987; Bertiaux et al., 2018), and it is currently unknown whether there is a restricted access of soluble components to the locked old flagellum (Bertiaux et al., 2018). When cytokinesis was inhibited in CEP164C-depleted cells using teniposide, the new flagellum grew longer. Although these results are not altogether incompatible with a balance point model, it does not easily explain how assembled flagella are maintained even in the absence of IFT.

Our results show that CEP164C is only acquired in the third cell cycle after initial basal body formation, and this coincides with completion of flagellum growth. This is consistent with CEP164C contributing to the locking mechanism at the entry point of flagellum close to the transition fiber area. This apparent “age discrimination” is not understood but could be via modifications to the axoneme to prevent disassembly, distal tip cap modifications, control at the base of the flagellum base, or a combination of these factors (Bertiaux and Bastin, 2020). CEP164C depletion appeared to be sufficient for unlocking growth in an already assembled flagellum, showing that control at the base of an assembled flagellum is important. Future work to discover candidate interacting partners of CEP164C could help to understand how this protein is differentially targeted.

In mammalian cells, CEP164 is critical for centriole docking to the membrane via distal appendages (analogous to transition fibers) and recruits the serine/threonine protein kinase TTBKII to the centriole, promoting cilia growth by the removal of coiled-coil protein 10 (Graser et al., 2007; Čajánek and Nigg, 2014; Schmidt et al., 2012; Burke et al., 2014). Here, docking to the flagellar pocket membrane and flagellum growth still occurred in the absence of CEP164C. Trypanosomes have three nonidentical CEP164 proteins (Hodges et al., 2010), which all contain the conserved N-terminal WW domain and a C-terminal coiled-coil domain, but differ significantly from CEP164C (21–26% identity). Unlike CEP164C, CEP164A and CEP164B both localize to the basal body of both old and new flagella (Dang et al., 2017). No orthologues of TTBKII and coiled-coil protein 10 were identified in *Trypanosoma brucei* by reciprocal best blast, suggesting these pathways are regulated by different proteins or trypanosome CEP164 performs a distinct function to its mammalian orthologue. There are multiple CEP164 orthologues in *Paramecium, Tetrahymena, Giardia, Leishmania*, and *Trichomonas* (Hodges et al., 2010), indicating that CEP164 proteins potentially perform multiple functions in regulation of flagellum/cilium growth in eukaryotic organisms. CEP164C could be part of a common regulatory mechanism in many ciliated unicellular organisms that have to deal with multiple flagella within a cell and is an underappreciated mechanism of flagellum regulation.

## Materials and methods

### Parasites and culture conditions

SmOxP9 procyclic *T. brucei* were used for all experiments. These were derived from TREU 927 strain, expressing T7 RNA polymerase and tetracycline repressor (Poon et al., 2012). Cells were cultured in SDM79 media supplemented with Hemin and 10% fetal calf serum (Brun and Schönenberger, 1979) at 28°C and maintained at a density between 1 × 10^6^ and 1 × 10^7^ cells/ml. Analysis of growth was performed at a starting density of 2 × 10^6^ cells/ml and monitored daily using a Z2 Coulter Counter.

### Cell line generation

A reporter cell line was made expressing TbSAS6 (Tb927.9.10550) endogenously tagged with mScarlet as a basal body marker and for Western blot analysis. This was generated using the PCR tagging methodology (Dean et al., 2015). Endogenous tagging of CEP164C (Tb927.1.3560) with mNG was performed. A tagging construct encoding the Ty::mNG tag and blasticidin resistance was amplified from the pPOTv4 plasmid using long primers incorporating 80 nt of homology to CEP164C and its adjacent UTR (Dean et al., 2015; forward, 5′-ATT​TCT​ACG​CTC​ACG​CTA​TAT​TAC​TCT​CTT​TCT​TTC​CAT​TAG​AAA​GGT​CGA​CTC​ATA​GTC​GAA​ATT​TTT​TTT​TTG​AAG​CTG​TAT​AAT​GCA​GAC​CTG​CTG​C-3′; reverse, 5′-AGC​CAT​TTC​CCG​TAC​TCC​AGA​AGC​TCG​GTT​TCT​GAA​GGT​TCG​TAA​TTG​TCG​CTC​GTC​ACA​ACA​TCT​AGA​ACT​ACG​GAC​ATA​CTA​CCC​GAT​CCT​GAT​CC-3′).

The amplicon was transfected into SmOxP9 procyclic form *T. brucei* using program X-001 on a Nucleofector 2b (Lonza). The RNAi plasmid for the knockdown of CEP164C was generated using the pQuadra system (Inoue et al., 2005). Oligos were designed for the amplification of CEP164C using RNAit (forward, 5′-TCT​GAA​ACG​CAT​TTG​TCT​GC-3′; reverse, 5′-GCA​CAG​CAC​AGA​GGT​TGA​AA-3′; Redmond et al., 2003). The PCR product and the plasmids pQuadra 1 and pQuadra 3 were digested with BstXII. Then, the PCR product and the fragment released from pQuadra 1 were ligated into the pQuadra 3 backbone. Following sequencing confirmation, the plasmid was linearized with NotI, and 2 µg was transfected into the endogenously tagged CEP164C:mNG cell line. Doxycycline (1 µg/ml) was added to induce knockdown of CEP164C expression.

For ectopic expression of CEP164C::mNG, the reporter cell line expressing TbSAS-6 (Tb927.9.10550) endogenously tagged with mScarlet as a basal body marker was used (see above). Oligos for ectopic expression of CEP164C were designed (forward, 5′-TTA​ACT​AGT​ATG​TCC​GTA​GTT​CTA​GAT​G-3′; reverse, 5′-aaa​gga​tcc​TTA​ATG​AGC​CGA​TTT​ACC​C-3′) and used to amplify the CEP164C ORF from *T. brucei* wild-type genomic DNA. The PCR product was digested with SpeI and BamHI and ligated into the pDEX777 vector digested with SpeI and XbaI (Poon et al., 2012). This was necessary due to the XbaI sites present in the sequence of the Cep164c PCR amplicon. Following sequencing confirmation, the plasmid was linearized with NotI and 2 µg transfected into the TbSAS6:mScarlet basal body marker cell line. Doxycycline (1 µg/ml) was added to induce ectopic CEP164C expression.

To monitor IFT trains following CEP164C depletion, endogenous tagging of IFT52 (Tb927.10.14980) with mNG was performed as above in the CEP164C RNAi cell line.

### Fixed and live-cell microscopy

For whole-cell immunofluorescence, cells were washed three times with PBS and allowed to settle on poly-L-lysine–coated slides before fixing with 2% formaldehyde in PBS for 10 min and then washing for 10 min in PBS. For cytoskeleton preparations, cells were washed three times with PBS and allowed to settle on poly-L-lysine–coated slides. 0.5% Nonidet P-40 in PEME (100 mM Pipes·NaOH, pH 6.9, 2 mM EGTA, 1 mM MgSO_4_, and 100 nM EDTA) was added to the cells for 30 s, followed by fixation in −20°C methanol for 20 min. Cytoskeletons were rehydrated in PBS for 10 min. For immunofluorescence, slides were incubated with primary antibodies diluted in PBS with 0.1% BSA for 1 h at room temperature followed by three washes of 10 min. Primary antibodies used were mouse monoclonal L8C4 (1:200; Kohl et al., 1999), rat monoclonal YL1/2 (Kilmartin et al., 1982; 1:40; a kind gift from Professor Keith Gull, University of Oxford, Oxford, UK), and Mab25 (Pradel, 2006; 1:100; a kind gift from Professor Derrick Robinson, University of Bordeaux, Bordeaux, France). Secondary antibodies were diluted in PBS with 0.1% BSA was added to the slides for 45 min at room temperature. Slides were washed three times in PBS for 10 min, with DAPI (2 mg/ml) added to the penultimate wash. Slides were mounted with coverslips using antifade reagent (ProLong antifade; Invitrogen). For live-cell microscopy, cells were washed three times in PBS supplemented with 10 mM glucose and 46 mM sucrose (vPBS). In the second wash, DNA was stained using 10 µg/ml Hoechst 33342. After the third wash, cells were resuspended in PBS, settled onto glass slides, mounted with a coverslip, and imaged immediately.

Cells and cytoskeletons were observed and images collected using a Zeiss Axioimager Z2 microscope with a Hamamatsu ORCA-Flash 4.0 camera. A planAPO 100× NA 1.46 oil-immersion objective was used, and all images were acquired at room temperature. Images were processed using Image J. Brightness and contract was adjusted for the whole image where necessary for clarity of the image. To measure relative fluorescence intensity in the ectopic expression studies, all images were taken at the same exposure time of 30 ms. Fluorescence intensity was determined using FIJI (ImageJ) by measuring the mean gray value in a given area surrounding the basal bodies of the new flagellum. A background value from the anterior region of the cell was subtracted from the measured value in each cell. For live video microscopy of IFT trafficking, cells expressing mNG::IFT52 were deposited it on glass slide, covered with a coverslip, and observed directly with a Leica DMI4000 microscope equipped with a 100× NA 1.4 lens (Leica) at room temperature. Videos were acquired using an Evolve 512 electron-multiplying charge-coupled device camera (Photometrics), driven by the Metavue acquisition software (Molecular Probes). IFT trafficking was recorded in at 100 ms per frame for 30 s. Kymograph analysis was used to quantify anterograde IFT using KymographDirect (v2.1).

### Western blot analysis

Cells were heated at 100°C in Laemmli loading buffer (4% SDS, 20% glycerol, 120 mM Tris-HCl, pH 6.8, and 0.2% bromophenol blue) for 5 min. Protein from 10^6^ cells/lane were separated on a 10% SDS-PAGE gel, followed by a transfer to a polyvinylidene difluoride membrane using the Mini Trans-Blot system (Bio-Rad), according to the manufacturer’s protocol. The transferred membrane was blocked with 5% skimmed milk (Sigma-Aldrich) in TBS-T (50 mM Tris-HCl, 100 mM NaCl, pH 7.6, and 0.1% Tween-20) for 1 h. Mouse monoclonal antibodies: anti-TY (1:200; Bastin et al., 1996), L8C4, L13D6 (Kohl et al., 1999; 1:200), a kind gift of Professor Keith Gull (University of Oxford, Oxford, UK) were incubated with the membrane for 1 h. Three membrane washes were performed with TBS-T and incubated for 1 h with anti-mouse IgG coupled to HRP (Jackson Laboratory), diluted 1:10,000. The membrane was washed three times with TBS-T following secondary antibody incubation. For final detection of protein, the chemiluminescence reaction was performed with WesternBright Quantum HRP substrate (Advansta) according to the manufacturer’s protocol.

### Inhibition of cell division

Teniposide (SML0609; Sigma-Aldrich), a topoisomerase II inhibitor, was dissolved in DMSO and added to 1.5 × 10^6^ cell/ml culture of the endogenously tagged CEP164C:mNG or CEP164C RNAi cell line 24 h after induction of RNAi at a final concentration of 200 µM (Bertiaux et al., 2018). In the control flasks, the same volume of DMSO without teniposide was added. Samples were collected 24 h after teniposide treatment. CEP164C RNAi cells were treated with teniposide after 24 h of RNAi induction and collected 24 h after teniposide treatment. RNAi induction was maintained with tetracycline during the 24-h treatment with teniposide.

### Transmission EM

A final concentration of 2.5% glutaraldehyde (TAAB) was added to the culture suspension. Cells were pelleted and resuspended in a primary fixative containing 2.5% glutaraldehyde, 2% paraformaldehyde (Agar Scientific), and 0.1% tannic acid (TAAB) in 0.1 M phosphate buffer, pH 7.0 (Sigma-Aldrich). Cells were fixed for 2 h at room temperature. Pellets were washed with 0.1 M phosphate buffer (pH 7.0) and postfixed in 1% osmium tetroxide (Agar Scientific) in 0.1 M phosphate buffer (pH 7.0) for 1 h at room temperature. Samples were rinsed and stained en bloc for 40 min in 2% aqueous uranyl acetate (TAAB), dehydrated in an ascending acetone series (Fisher Scientific), and embedded in hard formulation Agar 100 resin (Agar Scientific). 70-nm thin sections were made and stained using lead citrate for 5 min, followed by three washes with MilliQ water. Images were captured using a Hitachi H-7650 transmission electron microscope.

### Scanning EM

Uninduced control cells and 96-h-induced CEP164C RNAi samples were collected and a final concentration of EM grade 2.5% glutaraldehyde in PBS (pH 7.2) was added to the media. Cells were then concentrated by centrifugation, and 2.5% glutaraldehyde in PBS (pH 7.2) was added to cells for 2 h at room temperature. Cells were settled on 13-mm glass coverslips and washed three times with ddH_2_O, followed by a serial dehydration in ETOH (30%, 60%, 70%, and 90%, 3 × 100%). Samples were critical-point dried according to the manufacturer’s instructions and coated with gold. Images were captured using a Hitachi S-3400 microscope.

### Flagella measurements

Measurements of flagellum length were performed using ImageJ on images of cytoskeletons labeled with either the anti-axoneme antibody mAb-25 or the anti-PFR antibody L8C4. See above for details regarding the antibodies used.

### Statistics and graphs

The statistical tests, F test and Wilcoxon, were performed in RStudio. Pearson’s correlation test was calculated in Microsoft Excel. Graphs for the analysis were made in RStudio and Microsoft Excel. All errors correspond to the standard deviation of the population. Statistically significant differences are indicated (*, P < 0.05; **, P < 0.01; ***, P < 0.001). The number of samples analyzed for each experiment is indicated in the figure legends.

### Online supplemental material

Fig. S1 shows the results of EM of the CEP164C RNAi cell line.

## References

[bib1] Bastin P., Bagherzadeh Z., Matthews K.R., and Gull K. 1996 A novel epitope tag system to study protein targeting and organelle biogenesis in Trypanosoma brucei. Mol. Biochem. Parasitol. 77:235–239. 10.1016/0166-6851(96)02598-4 8813669

[bib2] Bastin P., Sherwin T., and Gull K. 1998 Paraflagellar rod is vital for trypanosome motility. Nature. 391:548 10.1038/35300 9468133

[bib3] Bertiaux E., and Bastin P. 2020 Dealing with several flagella in the same cell. Cell. Microbiol. 22:e13162 10.1111/cmi.13162 31945244

[bib4] Bertiaux E., Morga B., Blisnick T., Rotureau B., and Bastin P. 2018 A Grow-and-Lock Model for the Control of Flagellum Length in Trypanosomes. Curr. Biol. 28:3802–3814.e3. 10.1016/j.cub.2018.10.031 30449671

[bib5] Broekhuis J.R., Leong W.Y., and Jansen G. 2013 Regulation of cilium length and intraflagellar transport. Int. Rev. Cell Mol. Biol. 303:101–138. 10.1016/B978-0-12-407697-6.00003-9 23445809

[bib6] Brun R., and Schönenberger 1979 Cultivation and in vitro cloning or procyclic culture forms of Trypanosoma brucei in a semi-defined medium. Short communication. Acta Trop. 36:289–292. 43092

[bib7] Burke M.C., Li F.-Q., Cyge B., Arashiro T., Brechbuhl H.M., Chen X., Siller S.S., Weiss M.A., O’Connell C.B., Love D., 2014 Chibby promotes ciliary vesicle formation and basal body docking during airway cell differentiation. J. Cell Biol. 207:123–137. 10.1083/jcb.201406140 25313408 PMC4195830

[bib8] Čajánek L., and Nigg E.A. 2014 Cep164 triggers ciliogenesis by recruiting Tau tubulin kinase 2 to the mother centriole. Proc. Natl. Acad. Sci. USA. 111:E2841–E2850. 10.1073/pnas.1401777111 24982133 PMC4104846

[bib9] Craft J.M., Harris J.A., Hyman S., Kner P., and Lechtreck K.F. 2015 Tubulin transport by IFT is upregulated during ciliary growth by a cilium-autonomous mechanism. J. Cell Biol. 208:223–237. 10.1083/jcb.201409036 25583998 PMC4298693

[bib10] Dang H.Q., Zhou Q., Rowlett V.W., Hu H., Lee K.J., Margolin W., and Li Z. 2017 Proximity Interactions among Basal Body Components in Trypanosoma brucei Identify Novel Regulators of Basal Body Biogenesis and Inheritance. MBio. 8:e02120-16 10.1128/mBio.02120-16 28049148 PMC5210500

[bib11] Dean S., Sunter J., Wheeler R.J., Hodkinson I., Gluenz E., and Gull K. 2015 A toolkit enabling efficient, scalable and reproducible gene tagging in trypanosomatids. Open Biol. 5:140197 10.1098/rsob.140197 25567099 PMC4313374

[bib12] Dean S., Sunter J.D., and Wheeler R.J. 2017 TrypTag.org: A Trypanosome Genome-wide Protein Localisation Resource. Trends Parasitol. 33:80–82. 10.1016/j.pt.2016.10.009 27863903 PMC5270239

[bib13] Farr H., and Gull K. 2009 Functional studies of an evolutionarily conserved, cytochrome b5 domain protein reveal a specific role in axonemal organisation and the general phenomenon of post-division axonemal growth in trypanosomes. Cell Motil. Cytoskeleton. 66:24–35. 10.1002/cm.20322 19009637

[bib14] Goehring N.W., and Hyman A.A. 2012 Organelle growth control through limiting pools of cytoplasmic components. Curr. Biol. 22:R330–R339. 10.1016/j.cub.2012.03.046 22575475

[bib15] Gonçalves J., and Pelletier L. 2017 The Ciliary Transition Zone: Finding the Pieces and Assembling the Gate. Mol. Cells. 40:243–253. 10.14348/molcells.2017.0054 28401750 PMC5424270

[bib16] Graser S., Stierhof Y.-D., Lavoie S.B., Gassner O.S., Lamla S., Le Clech M., and Nigg E.A. 2007 Cep164, a novel centriole appendage protein required for primary cilium formation. J. Cell Biol. 179:321–330. 10.1083/jcb.200707181 17954613 PMC2064767

[bib17] Hodges M.E., Scheumann N., Wickstead B., Langdale J.A., and Gull K. 2010 Reconstructing the evolutionary history of the centriole from protein components. J. Cell Sci. 123:1407–1413. 10.1242/jcs.064873 20388734 PMC2858018

[bib18] Inoue M., Nakamura Y., Yasuda K., Yasaka N., Hara T., Schnaufer A., Stuart K., and Fukuma T. 2005 The 14-3-3 proteins of Trypanosoma brucei function in motility, cytokinesis, and cell cycle. J. Biol. Chem. 280:14085–14096. 10.1074/jbc.M412336200 15653691

[bib19] Jiang L., Wei Y., Ronquillo C.C., Marc R.E., Yoder B.K., Frederick J.M., and Baehr W. 2015 Heterotrimeric kinesin-2 (KIF3) mediates transition zone and axoneme formation of mouse photoreceptors. J. Biol. Chem. 290:12765–12778. 10.1074/jbc.M115.638437 25825494 PMC4432293

[bib31] Kilmartin J.V., Wright B., and Milstein C. 1982 Rat monoclonal antitubulin antibodies derived by using a new nonsecreting rat cell line. J. Cell Biol. 93:576–582. 6811596 10.1083/jcb.93.3.576 PMC2112140

[bib20] Kohl L., and Bastin P. 2005 The flagellum of trypanosomes. Int. Rev. Cytol. 244:227–285. 10.1016/S0074-7696(05)44006-1 16157182

[bib21] Kohl L., Sherwin T., and Gull K. 1999 Assembly of the paraflagellar rod and the flagellum attachment zone complex during the Trypanosoma brucei cell cycle. J. Eukaryot. Microbiol. 46:105–109. 10.1111/j.1550-7408.1999.tb04592.x 10361731

[bib22] Marshall W.F., and Rosenbaum J.L. 2001 Intraflagellar transport balances continuous turnover of outer doublet microtubules: implications for flagellar length control. J. Cell Biol. 155:405–414. 10.1083/jcb.200106141 11684707 PMC2150833

[bib23] Marshall W.F., Qin H., Rodrigo Brenni M., and Rosenbaum J.L. 2005 Flagellar length control system: testing a simple model based on intraflagellar transport and turnover. Mol. Biol. Cell. 16:270–278. 10.1091/mbc.e04-07-0586 15496456 PMC539171

[bib24] Poon S.K., Peacock L., Gibson W., Gull K., and Kelly S. 2012 A modular and optimized single marker system for generating Trypanosoma brucei cell lines expressing T7 RNA polymerase and the tetracycline repressor. Open Biol. 2:110037 10.1098/rsob.110037 22645659 PMC3352093

[bib32] Pradel L.C. 2006 NIMA-related kinase TbNRKC is involved in basal body separation in Trypanosoma brucei. J. Cell Sci. 119(9):1852–1863. 16608878 10.1242/jcs.02900

[bib25] Redmond S., Vadivelu J., and Field M.C. 2003 RNAit: an automated web-based tool for the selection of RNAi targets in Trypanosoma brucei. Mol. Biochem. Parasitol. 128:115–118. 10.1016/S0166-6851(03)00045-8 12706807

[bib26] San Agustin J.T., Pazour G.J., and Witman G.B. 2015 Intraflagellar transport is essential for mammalian spermiogenesis but is absent in mature sperm. Mol. Biol. Cell. 26:4358–4372. 10.1091/mbc.E15-08-0578 26424803 PMC4666132

[bib27] Schmidt K.N., Kuhns S., Neuner A., Hub B., Zentgraf H., and Pereira G. 2012 Cep164 mediates vesicular docking to the mother centriole during early steps of ciliogenesis. J. Cell Biol. 199:1083–1101. 10.1083/jcb.201202126 23253480 PMC3529528

[bib28] Schneider A., Sherwin T., Sasse R., Russell D.G., Gull K., and Seebeck T. 1987 Subpellicular and flagellar microtubules of Trypanosoma brucei brucei contain the same alpha-tubulin isoforms. J. Cell Biol. 104:431–438. 10.1083/jcb.104.3.431 3818788 PMC2114526

[bib29] Slaats G.G., Ghosh A.K., Falke L.L., Le Corre S., Shaltiel I.A., van de Hoek G., Klasson T.D., Stokman M.F., Logister I., Verhaar M.C., 2014 Nephronophthisis-associated CEP164 regulates cell cycle progression, apoptosis and epithelial-to-mesenchymal transition. PLoS Genet. 10:e1004594 10.1371/journal.pgen.1004594 25340510 PMC4207587

[bib30] Stephan A., Vaughan S., Shaw M.K., Gull K., and McKean P.G. 2007 An essential quality control mechanism at the eukaryotic basal body prior to intraflagellar transport. Traffic. 8:1323–1330. 10.1111/j.1600-0854.2007.00611.x 17645436

